# Double Bolus Application in TWIST-MR-Angiography of the Cervical Arteries

**DOI:** 10.1155/2012/203538

**Published:** 2012-10-18

**Authors:** Andreas Korn, Michael Fenchel, Till-Karsten Hauser, Sotirios Bisdas, Thomas Nägele, Ulrike Ernemann, Uwe Klose, Benjamin Bender

**Affiliations:** Department of Diagnostic and Interventional Neuroradiology, University of Tübingen, Hoppe-Seyler Street 3, 72076 Tübingen, Germany

## Abstract

*Purpose*. The aim of the present work was to test the feasibility of the time-resolved MR-angiography (TWIST-MRA) of cervical arteries using double bolus injection. *Material and Methods*. TWIST-MRA with a temporal resolution of 8.4 seconds for each frame and a spatial resolution with a voxel size of 0.61 × 0.58 × 0.8 mm^3^ was performed in 24 patients. A biphasic bolus injection protocol was used with the second injection being started 21 seconds after the first contrast dye bolus. Diagnostic image quality was rated according to a 4-point scale. *Results*. In 12 patients (50%) no clear separation between the cervical venous and arterial vessels was evident after the first bolus injection. Using TWIST-MRA data acquired after the second bolus a sufficient diagnostic image quality (rating ≥3, mean 3.5) could be obtained in 22 of 24 patients (92%). *Discussion*. The double bolus injection protocol using TWIST-MRA allows for very good separation of the cervical arteries.

## 1. Introduction

Conventional 3D contrast-enhanced magnetic resonance angiography (3D-CE-MRA) is used for the assessment of the cervical arteries in routine clinical settings [1]. This technique requires exact timing of contrast injection and data acquisition to obtain selective arterial contrast for the assessment of the arterial tree. The quality of 3D-CE-MRA images depend strongly on a sufficiently high concentration of contrast agent in the arteries while acquiring central k-space data. Only if data acquisition of central k space coincides with arrival of the contrast medium in cervical arteries, images with selective arterial contrast without venous contamination are acquired [2]. In patients with variable bolus arrival time, for example, due to cardiac arrhythmia, this can be challenging.

Recently, 3D-time-resolved-MRA (3D-TR-MRA) techniques were applied to the cervical arteries using a combination of parallel imaging (generalized autocalibrating partially parallel acquisition, GRAPPA) [3, 4], and time-resolved imaging with stochastic trajectories (TWIST-MRA) [5]. Imaging with TWIST-MRA is based on a view-sharing technique, which undersamples the periphery of k space depending on the radial distance from the center of k space [6] and combines anatomic and hemodynamic image information [7].

The goal of the present work was to establish the benefit of using a double bolus injection protocol for the TWIST-MRA of the cervical vessels.

## 2. Materials and Methods

### 2.1. Patients

The study was conducted in accordance with the local IRB.

TWIST-MRA was performed in 24 patients (10 men and 14 women; age range: 33–52 years; mean age: 43 ± 14 years) who were scheduled for contrast-enhanced cerebral MRI. In seven patients (3 men and 4 women; age range: 27–66 years: mean age 50 ± 15 years) a standard TWIST MRA with high temporal resolution was conducted as a reference.

### 2.2. MR Imaging

All examinations were performed on a 32-channel 1.5-T whole-body MR scanner equipped with commercially available 8 channel head and 4 channel neck matrix coils (Magnetom Avanto, Siemens Medical Solutions, Erlangen, Germany). The imaging volume was placed in a coronal direction to include the carotid and vertebral arteries from the aortic arch to the skull base. TWIST-MRA was applied using a 3D fast gradient-recalled echo (GRE) sequence with the following parameters: TR/TE 3.37 ms/1.33 ms, flip angle 25°, bandwidth 660 Hz/pixel, FOV 260 × 260 mm², image matrix 426 × 448, and slice thickness 0.8 mm (voxel size 0.61 × 0.58 × 0.8 mm³). The percentage of the center of k space (A), compared with the entire k-space volume that is sampled with every acquisition (controlling image contrast), and the percentage of data points in the periphery of k space (B) sampled per acquisition, was set to A = 15% and B = 25%. GRAPPA with an acceleration factor of 2 was used. By combining GRAPPA and TWIST, the resulting 3D data sets were acquired with a temporal resolution of 8.4 seconds for each frame with a total of 15 sequential measurements (temporal footprint 23.2 seconds).

For comparison a low spatial resolution TWIST-MRA (FOV 330 × 330 mm², voxel size 1.2 × 1.0 × 3.0 mm³, A = 15%, B = 25%) was used with a temporal resolution of 1,28 seconds, a temporal footprint of 3.5 seconds, and 45 sequential measurements.

### 2.3. Contrast Agent Injection Protocol

**Figure 1:**
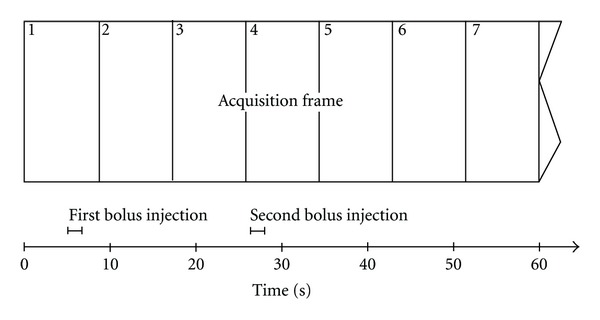
Timing of image acquisition: there is a 21 s (exactly 2.5 times the acquisition time frame) interval between start of the first and second bolus application. The solid and dashed lines schematize the contrast agent volume in the arterial and venal vessels. In the depicted case the second bolus would yield optimal contrast (see Figure 2 bottom row).

An electronic MR injection system (Spectris; Medrad, USA) was used for a biphasic injection protocol: 5 mL of Gadobutrol (Gadovist; Bayer Schering Pharma, Berlin, Germany) were injected at a flow rate of 3 mL/s beginning 5 seconds after start of data acquisition (the first image was therefore precontrast). The second injection of 5 mL Gadobutrol was started with a delay of 21.0 seconds after the first injection automatically, which is the length of two and a half measurement frames (Figure 1). Therefore the second injection started nearly at the beginning of the fourth sequential measurement. Each contrast agent application was followed by a saline flush of 20 mL with a flow rate of 3.0 mL/s. For the low spatial resolution TWIST-MRA the second injection bolus was omitted.

### 2.4. Postprocessing and Image Evaluation

Postprocessing was performed with scanner software (Syngo, Siemens) using subtracted MR-angiographic data to allow for reconstruction of multiplanar projection reformats (MPR) and maximum intensity projections (MIP) of TWIST.

Image evaluation was performed by two experienced neuroradiologists in a consensus reading. Image quality of the respective 3D datasets was rated according to a 4-point scale: 1 = no arterial enhancement, nondiagnostic; 2 = minimal arterial enhancement, marginal confidence in diagnostic content; 3 = diagnostic; 4 = maximal arterial enhancement, high confidence in diagnosis.

In two cases a high resolution 3D-CE-MRA and a conventional angiography were available for comparison.

## 3. Results

In 10 of 24 patients (42%) sufficient depiction (rating ≥ 3 of the arterial vessels in one time frame) was achieved with the first bolus whereas in 14 patients (58%) no clear separation between the venous and arterial vessels was evident.

**Figure 2:**
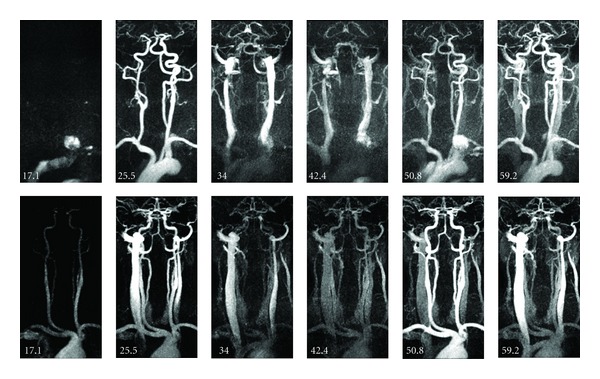
Top row: MR-angiogram with optimal arterial contrast after first bolus (*t* = 25.5 s). Bottom row: MR-angiogram with optimal arterial contrast after second bolus (*t* = 50.8 s). Note that the first time frame is not depicted.

**Figure 3:**
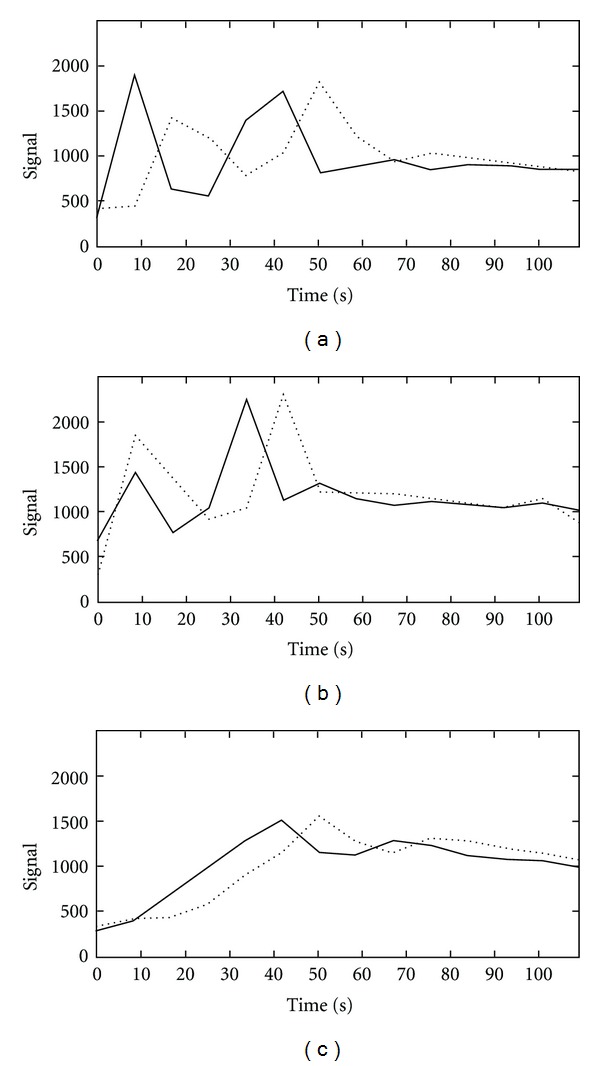
Mean signal curves over time for ROIs positioned in the carotic bifurcation (solid lines) and ROIs placed in the jugular vein (dashed lines) for the patients shown in Figure 2 top row (a) and bottom row (b) and a patient with cardiovascular disease and extreme bolus dispersion (c).

In 12 of these 14 cases, the second bolus yielded sufficient arterial enhancement and confident assessment of the arteries was possible.  Figure 2 depicts typical TWIST-MR-angiograms obtained with the first and second bolus. Figures 3(a) and 3(b) depict the mean value curves over time from the carotic bifurcation and jugular vein for two examples shown in Figure 2. Venous contamination as a result of the first bolus, in patients requiring a second bolus for sufficient arterial enhancement, did not influence the diagnostic value when multiplanar or maximum-intensity reconstructions were postprocessed.

Including the data acquired after the second bolus, a sufficient diagnosis (rating ≥ 3, mean 3.5) could be obtained in 22 of 24 patients (92%).

The two patients with insufficient results in all images (rating 1 and 2) had unusual long circulation time due to cardiopulmonary diseases, as the enhanced bolus dispersion led to consecutively broadening of the arterial signal peak (Figure 3(c)). In both cases a static 3D-CE-MRA yielded no diagnostic images, too.

**Figure 4:**
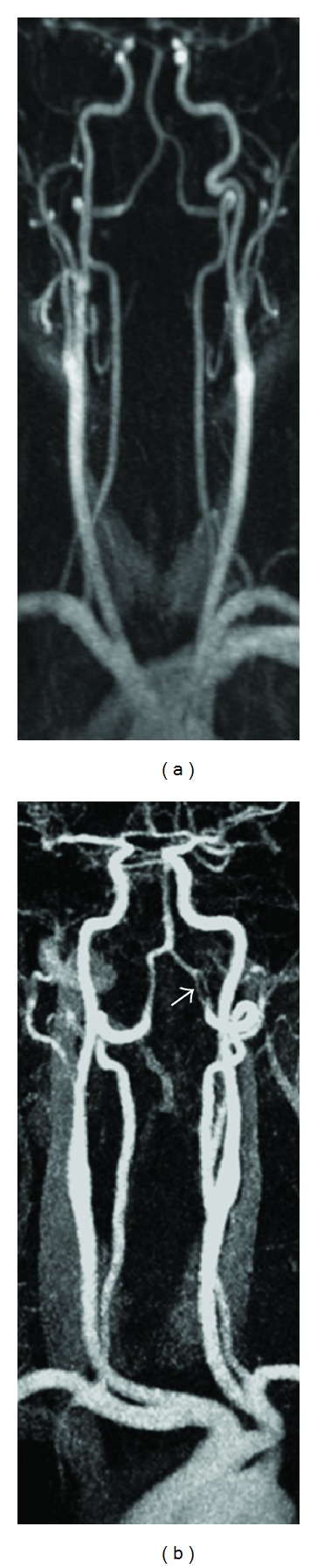
Comparison of a standard high temporal and low spatial resolution TWIST-MRA (a) and a high spatial resolution TWIST MRA (b) in two different patients. Due to the lower spatial resolution the vessels in (a) appear blurred and vascular anomalies like stenosis or fenestration (e.g., arrow in b) might be missed.

**Figure 5:**
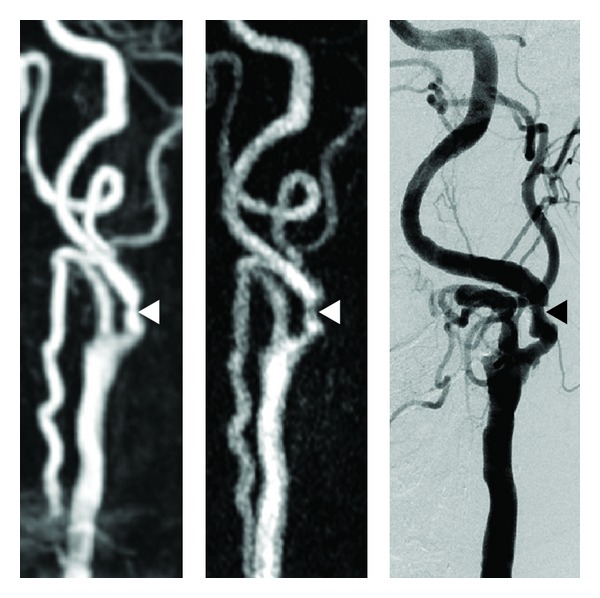
From left to right, conventional 3D-CE-MRA, TWIST-MRA obtained with the first bolus and DSA depicting a symptomatic and therefore hemodynamic relevant short stenosis of the left internal carotid artery.

In 7 out of 7 control cases, a sufficient arterial enhancement was obtained (Figure 4). In both patients with additional 3D-CE-MRA and conventional angiography high-spatial resolution TWIST-MRA identification and evaluation of stenosis was possible (Figure 5).

## 4. Discussion

For a measurement time of just several seconds, optimal bolus timing is vital for 3D-CE-MRA. If central k-space data are acquired too early, ringing or banding artifacts are generated [8]. Conversely, if central k-space data acquisition is too late, diminished arterial signal intensity as well as venous contamination is the consequence.

In contrast, the TWIST-MRA uses a shorter measurement time for a single frame and acquisition of several subsequent frames. These accounts for that arterial and venous signal peaks are in different frames. So, the start of the measurement is no more dependent on the skill level of the operator. It enables the evaluation of dynamic parameters, which may help identifying hemodynamic changes, like delayed filling of the carotid bulb in carotid dissection, delayed opacification of a distal cervical internal carotid dissection following opacification of the circle of Willis, or retrograde filling of a vertebral artery distal to an occlusion [5]. Associated venous diseases, such as arteriovenous fistulas, can be also optimally displayed [5]. To obtain a high temporal resolution usually low spatial resolution protocols are used, that have a decreased sensitivity for the identification of arterial stenosis.

Our study shows that TWIST-MR-angiograms with high spatial resolution (voxel size 0.6 × 0.6 × 0.8 mm³) yield a good separation of arteries and veins in 92% of all patients, if a double bolus injection protocol is used to account for the lower temporal resolution. The high spatial resolution should enable the evaluation of stenosis, which was shown in two patients (Figure 5). An injection interval of 21.0 seconds between the first and second bolus is according to our experience recommended, as earlier injection of the second bolus results in more disturbing superposition of arteries and veins. By starting the second bolus with an interval of 21.0 seconds after the first bolus, sampling of the center of k space is shifted by half of the frame duration in comparison to the start of the bolus injection. This improves the probability to measure at least one frame with high arterial signal and low venous signal. Therefore the second bolus injection successfully compensates the loss of temporal resolution. Correspondingly, in 50% of all cases best arterial contrast was achieved with the second bolus. In some of these patients the previous bolus was accompanied by a small venous contamination of the images. While diagnostic evaluation was not compromised, a complete elimination of the veins signal is possible to further increase image quality by subtraction of a previous measurement with selective venous contrast.

There are several limitations to this study. First the long temporal footprint together with the short bolus can cause variable signal intensities within the vessels at bolus arrival that can alter the dynamic information, although the specific design of the syngo TWIST trajectories ensures that spatial resolution information is provided even with only one B trajectory (direct communication with Siemens). Additionally Edge Enhancement artifacts can occur at the bolus wash out. Both artifacts were not observed in our study and should not alter image quality in the frame with maximum arterial contrast. A slower injection rate could help to further limit these two artifacts, but could also increase venous contamination in the frame with maximum arterial contrast.

This study was limited to a cohort of rather young patients and further studies should include elderly patients too. In these patients a higher degree of atherosclerosis and divergence of hemodynamic parameters can be expected, which might negatively influence image quality. But especially in the patients with a high divergence of hemodynamic parameters TWIST-MRA could prove useful, although extreme bolus dispersion can still make the acquisition of diagnostic images impossible.

While in two patients with known vascular pathologies (carotid stenosis) the TWIST-MRA proved to provide a sufficient depiction of the stenosis in comparison to 3D-CE-MRA and conventional angiography, this has to be proven in a further study that compares the high resolution TWIST-MRA with a gold standard (3D-CE-MRA or conventional angiography) in a patient group with suspected or known vascular pathologies.

## 5. Conclusion

In conclusion high spatial resolution TWIST-MRA with double bolus injection provides acceptable image quality in the majority of patients and could be a useful alternative to conventional 3D-CE-MRA in patients with variable hemodynamic parameters due to the independence of correct bolus timing by the operator. In contrast to high temporal resolution TWIST-MRA the identification of stenosis seems to be possible at the cost of less dynamic information.
